# The Effect of Core-Hole Shape on Attosecond Valence Electron Dynamics

**DOI:** 10.1021/acs.jpca.5c01706

**Published:** 2025-08-13

**Authors:** Tai Hua, Lucas Kurkowski, Kenneth Lopata

**Affiliations:** † Department of Chemistry, 5779 Louisiana State University, Baton Rouge, Louisiana 70803, United States; ‡ Center for Computation and Technology, 5779 Louisiana State University, Baton Rouge, Louisiana 70803, United States

## Abstract

Rapid X-ray ionization of a core electron is known to induce the attosecond motion of valence electrons; however, the effect of core-hole shape on the triggered dynamics remains relatively unknown. In this work, the sub-four fs response of prototypical functionalized/heterocyclic/polycyclic molecules was simulated using real-time time-dependent density functional theory (RT-TDDFT), a sudden approximation core-hole, and phenomenologically Auger–Meitner (AM) decay. These molecules included fluorobenzene, chlorobenzene, bromobenzene, phenol, thiophenol, pyridine, phosphorus, and azulene. It is observed that the valence electron dynamics are essentially independent of the core-hole created, provided that it is ionized from an inner-shell orbital and not an inner-valence orbital. This has broad implications for free-electron laser studies of X-ray pumped attosecond processes since the flexibility in edge allows for a wide range of experimental modalities, core-holes with longer AM lifetimes, and molecular targets.

## Graphical abstract

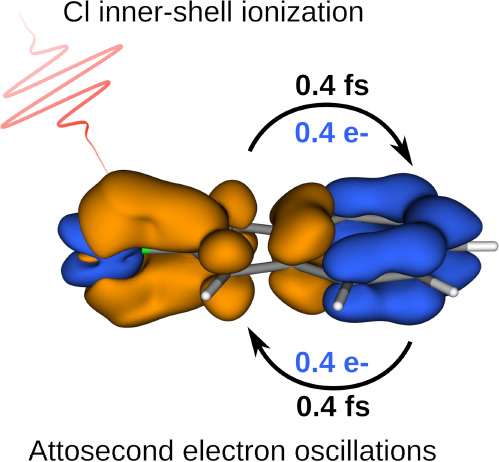

## 1 Introduction

With recent advancements in laser techniques, observing attosecond dynamics has become experimentally possible.^1–7^ This opens the door to understanding how electrons move coherently in molecules on time scales faster than nuclear motion occurs. An example of this phenomenon is charge migration, which was first predicted by Cederbaum and Zobeley in the late 1990s.^8^ Some hallmark experiments that have measured attosecond electronic processes include: valence dynamics in amino acids,^9^ charge migration in iodoacetylene,^10^ valence oscillations in N_2_ and CO_2_,^11^ core-hole induced attosecond valence dynamics in para-aminophenol,^12^ and the response of liquid water to an attosecond soft X-ray pump.^13^

There are a few modalities used to pump a system in attosecond studies. One way is to induce valence dynamics via direct pumping of the valence electrons in a molecule using UV/visible or XUV light.^14–17^ This has been extensively studied computationally, including neutral valence pumping with UV/visible laser pulses,^18–20^ or in the context of quantum control.^21,22^ Disadvantages of these schemes include the difficulty of using such pulses experimentally and of describing the pump step in an explicit manner. Instead of pumping, another approach is to ionize the valence such as using strong-field ionization (SFI),^10,23,24^ which has been computationally shown to result in charge migration in a wide range of molecules.^25–31^ SFI-induced processes, however, can also be difficult to probe experimentally, but in principle can be done such as using high harmonic spectroscopy.^10,31^ Ionizing from the inner-valence has also gained interest recently, where a UV or XUV field can be used to leave a molecule in a superposition of cationic states. Examples of this include XUV pumped attosecond dynamics in tryptophan^15^ and phenylalanine.^32^ Inner-valence pumping/ionization may be local or nonlocal, depending on the wavelength and system, which necessitates simulations on a case-by-case basis for prediction and interpretation.

Another possible modality is to use X-rays to create core-hole interactions that indirectly induce dynamics. In this type of process, the localized core-hole causes the valence to see a different potential, or equivalently, the rapid ionization leaves the molecule in a complicated superposition of cationic states. This has been shown computationally to result in long-range charge oscillations in the valence.^33–39^ A distinct advantage of using soft X-rays, in particular, is that the edges are element-specific, enabling pumping at one end of the molecule and probing at another, such as with X-ray transient absorption.^27,39–44^ Moreover, core-holes are somewhat universal in that they are not as molecule-specific as inner-valence and valence states. Given these, combined with recent developments in X-ray free-electron lasers, soft X-ray induced processes in molecules are rapidly gaining interest in the community.^12,13,45^ Despite its many advantages, the effect of different core-holes on induced valence dynamics remains relatively unknown. Some potentially important factors include: core-hole shape/localization/edge (e.g., 1s vs 2s), the element on which the core-hole is created (e.g., N vs O), the location of the heteroatom (e.g., attached to a ring or incorporated into the π system), and the role of Auger–Meitner decay.

To address some of these critical questions, in this paper, we present a systematic simulation study of attosecond valence electron dynamics induced by core-holes in benzene derivatives. Benzene derivatives have been shown to make good candidates for valence dynamics studies^25,29^ and form the basis for more complex organic molecules. Furthermore, benzene derivatives have been predicted to have interesting attosecond hole dynamics while using a frozen nuclei approximation,^29,33,46^ and benzene itself has been predicted to support hole motion that survives nuclear motion.^47^ One goal of this work is to help in the design of future free-electron laser soft X-ray pumped experiments, specifically to identify which X-ray edges to use.

As discussed below, for our simulations, we use real-time time-dependent density functional theory (RT-TDDFT) with the B3LYP global hybrid functional and a sudden ionization approximation. Regarding the validity of this method, although it has not been conclusively established, TDDFT has been shown to perform qualitatively similar to wave function methods (CAS-SCF, CCSD) in numerous studies of dynamics in molecules.^48^ It has also been shown to capture charge migration accurately as validated versus high harmonic spectroscopy experiments for bromo/iodoacetylene,^25^ CO_2_ and N_2_,^11^ and butadiyne.^49^ Uncertainty remains, however, about the general ability of RT-TDDFT to capture the attosecond dynamics. For example, it was shown by Kuleff and Dreuw that TDDFT fails when using sudden ionization out of a valence molecular orbital (MO).^50^ In this case, self-interaction errors in the exchange-correlation functional manifest as unphysical orbital delocalization and thus spurious dynamics. This can be remedied by using constrained density functional theory which uses a multiorbital energy minimization process,^51^ which has been shown to give TDDFT charge migration dynamics comparable to experimentally measured ones for the case of valence holes in halogenated acetylene.^25^ Another limitation of TDDFT is that in a linear response form (LR-TDDFT), it cannot obtain satellite (e.g., shakeup) peaks.^52^ This is a consequence of LR-TDDFT being a single excitation theory,^53,54^ which is related to the adibatic approximation (lack of memory in the exchange-correlation functional). This can be sidestepped by using a specially prepared core-hole initial state, such as equivalent core hole (ECH) LR-TDDFT.^55,56^ For an overview of methods for computing core spectroscopy see ref 57.

In this study, however, we use a sudden core-hole ionization and real-time propagation from this nonstationary state, so the single excitation limitation of linear response TDDFT does not obviously apply. RT-TDDFT, for example, has been shown to give nonlinear optical properties^58^ and excited state absorption when propagated from a nonstationary initial state.^59^ Indeed, TDDFT using a sudden core-hole has been previously shown to match ADC(4) for N K-edge induced valence dynamics in nitrosobenzene, which is a process related to a shakeup peak.^33^ Given these collective uncertainties, another goal of this work is to supply much-needed attosecond simulation results regarding core-hole induced dynamics. The broad range of systems and core-holes presented here should make the results valuable for comparing against other simulation methods and ultimately experiment. As a first step in this direction, in this paper, we also present a TDDFT validation study. In lieu of experimental dynamics results, which have yet to be reported, we instead compare the sudden core-hole TDDFT against experimental X-ray photoelectron (XPS) satellite energies. Since these states are known to be involved in the valence dynamics,^33,34^ the shakeup energies are a decent proxy for validating the accuracy of a method for the associated dynamics.

The remainder of the paper takes the following form. In Section 2 we briefly describe the simulation methodology; in Section 3 we present simulations of core-hole induced valence dynamics in a range of molecules/heteroatoms/X-ray edges; and in Section 3.5 we validate our method against experimental XPS satellite peak energies. The main conclusions are presented in Section 4.

## 2 Methods

All simulations used the RT-TDDFT module^60^ in NWChem.^61^ The geometries were optimized in the gas phase using the def2-TZVP basis and the B3LYP functional. The subsequent RT-TDDFT simulations used the B3LYP functional and the def2-TZVP basis for carbon and hydrogen and def2-QZVP for heteroatom. See the SI for basis set convergence information. To emulate an initial core-hole, we used a sudden approximation where one electron was removed from a particular molecular (generally atomic-like) orbital of the neutral ground state molecule without a subsequent energy minimization. This initial state results in numerous cationic states being populated. Multiexcitation TDDFT initial conditions like this have been used previously for core-triggered dynamics^33^ and valence hole electron dynamics.^25,51,62^ For time-integration, we used a second-order Magnus (exponential midpoint) predictor-corrector scheme with a time step of 0.2 a.u. (0.005 fs). The nuclei are frozen during the TDDFT simulation, which is a reasonable approximation given the short (≤3 fs) time scales. We neglect spin–orbit (SO) coupling, which is potentially important for the fourth row and lower elements. Given the observed insensitivity of the dynamics to core-hole shape, however, we expect that an L_2_ versus L_3_ core-hole will give similar dynamics. As an improvement, SO coupling can be incorporated via relativistic real-time TDDFT formalisms, which have been successful for X-ray absorption spectra.^63–66^

To analyze the dynamics, the time-dependent number of electrons was computed on particular regions of the molecule, *n*_R_(*t*) = ∫_R_d*V*ρ­(**r**,*t*), where *R* is the region and ρ­(**r**,*t*) is the electron density. The density was calculated on a Cartesian grid with a resolution of 0.035 Å^–3^ and a minimum 3.0 Å buffer in each direction from the position of each atom. See SI for information regarding convergence with respect to the density grid resolution. We report the deviation of the charge from the average Δ*n*_R_(*t*) ≡ *n*_R_(*t*) – ⟨*n*_R_(*t*)⟩, which better shows the charge oscillations for times after attosecond relaxation. When the Auger–Meitner (AM) decay time is similar to or less than to the dynamical period it can substantially limit the observability of the electron dynamics.^34,67^ Alternatively, AM decay itself can induce dynamics.^36^ RT-TDDFT poorly describes Auger–Meitner decay,^68^ and thus instead we use a phenomenological damping of the time-dependent charges to give a rough measure of how important AM decay would be for a particular process. We damp Δ*n*_R_(*t*) with an exponential e^–*t*/τ^, where τ is the literature computed mean decay lifetime for each particular core-hole.^69^ Where applicable, we use the L_2_ or M_2_ lifetimes for 2p and 3p core-holes, respectively. The details and numerical values are given in Table S2 in the SI.

## 3 Results

We focus on dynamics induced by a sudden ionization from a single core orbital for four classes of molecules: halobenzenes, phenol analogs, pyridine analogs, and chloroazulene (a fused five/seven membered ring). Figure 1 shows the structure of these molecules. This selection of molecules was chosen to represent a range of common benzene derivatives as well as a selection of heteroatoms across and down the periodic table. By going down the periodic table, the shape of the various core-holes changes; there are more inner-shell states available to ionize from, and there is the possibility of creating highly localized core-holes.

**Figure 1:**
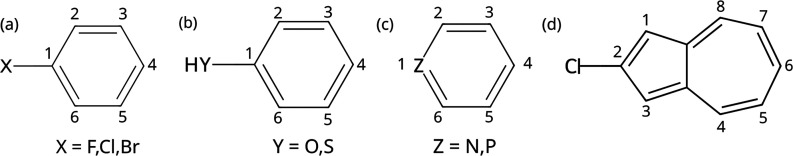
Structures of the molecules studied: (a) halogenated benzenes, (b) phenol and thiophenol, (c) pyridine and phosphorine, and (d) 2-chloroazulene.

### 3.1 Halobenzenes

First, we discuss the halobenzenes (X-C_6_H_5_, X = F, Cl, Br). This family of molecules represents the case where the core-hole site (halogen) is not integrated into the ring. Before going into the systematics, we first describe chlorobenzene in detail. Figure 2 shows the time-dependent change in electron number on three regions of the molecule (around Cl, left half of ring, right half of ring) for four different types of initial ionization. This figure shows both the undamped (a–c) and AM-damped dynamics (d–f). The purple curves in Figure 2 show the number of electrons on the chlorine atom following Cl 1s core-hole ionization. As shown in panel (d), the Cl rapidly (∼100 as) gains ∼0.5 of an electron as the valence density responds to the sudden change in the potential near the Cl atom. Looking at the change in number of electrons on the left half (Δ*n*_L_(*t*)) of the ring (e) and on the right half (Δ*n*_R_(*t*)) of the ring (f) shows that this results from a density of electron flow from the ring to the Cl. This few-hundred attosecond process is essentially the universal ionization dynamics time scale first reported by Breidbach and Cederbaum.^70^ Identical ∼100 as dynamics occurs for Cl 2s (blue) and Cl 2p holes (red), whereas the Cl 3s hole is different (discussed below).

**Figure 2:**
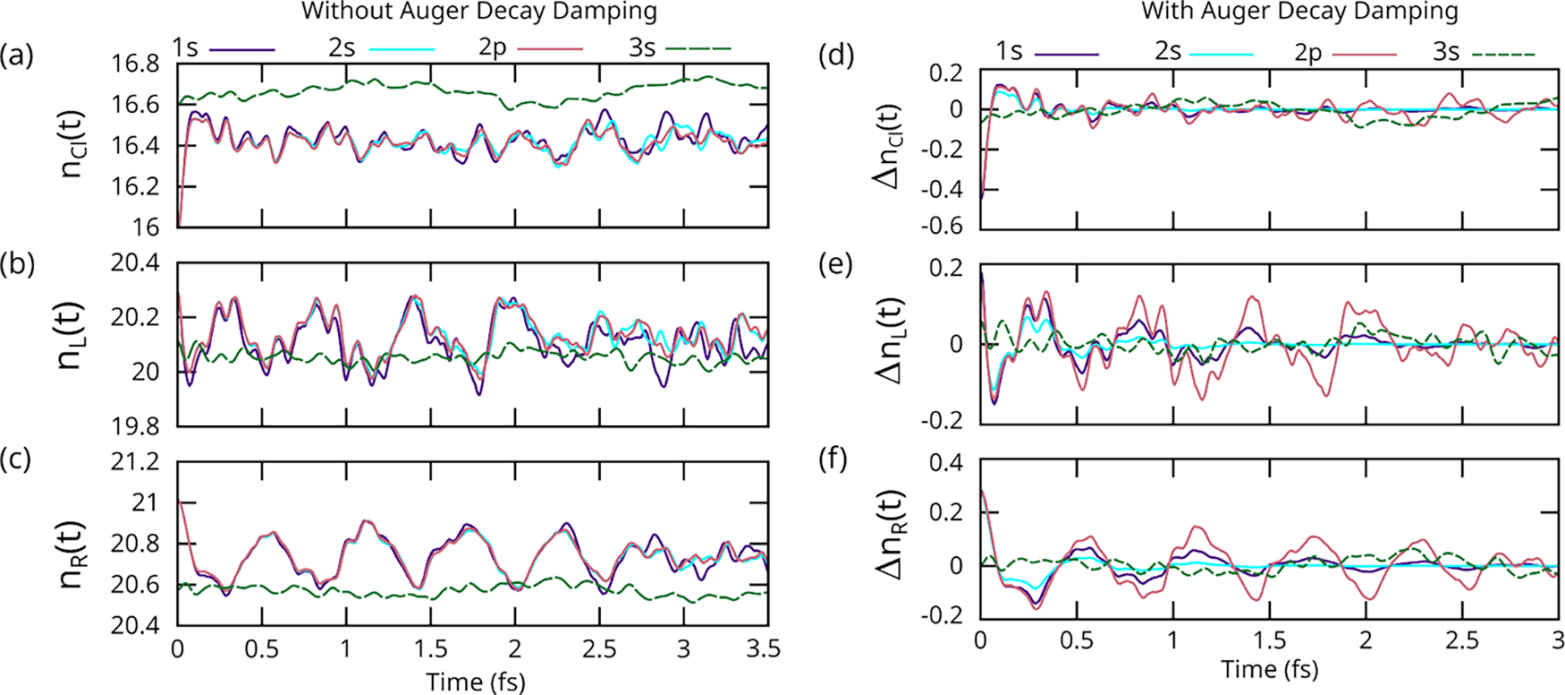
Time-dependent change in the number of electrons on three regions of chlorobenzene following different core-hole ionization triggers: (a) around the Cl, (b) on the left half of the benzene ring, and (c) on the right half of the ring. On each plot, solid lines denote inner-shell ionization, and dashed-lines denote inner valence. (d), (e), and (f) Same data but with the average subtracted and the difference number damped with the Auger–Meitner decay time associated with the core-hole.

After this attosecond response, the molecule exhibits femtosecond-time-scale electron motion. Looking at the Cl 1s core-hole case, loosely speaking, there are two dominant frequencies. There is a high frequency oscillation period (*T* ∼ 0.19 fs) that involves the chlorine atom and the left side of the ring. This is caused by the rapid motion of valence electrons between the left half of the ring and the valence region above the static core-hole. These modes appear as minor contributions to the charge on the right half of the ring. There is also a low frequency mode (*T* ∼ 0.565 fs) that involves the left and right halves of the ring. This is caused by the sloshing of electrons between the left and right side of the ring, as shown by the anticorrelation between the slow dynamics in Δ*n*_L_(*t*) and Δ*n*_R_(*t*) in Figure 2e,f. These K-edge triggered dynamics persist for approximately four oscillations before damping out due to the relatively fast τ = 1.2 fs Cl 1s AM decay.

The 1s (purple) and 2p_*y*_ (red) Δ*n*_R_(*t*) in Figure 2 holes give strikingly similar charge dynamics (period ∼ 2.5 fs), albeit with the 2p_*y*_ core-hole showing more persistent oscillations due to its longer AM decay time of 6.2 fs. Since the dynamics resulting from Cl 2p_*x*_, 2p_*y*_, and 2p_*z*_ core-holes are observed to be all essentially identical (see SI), we henceforth use a 2p_*y*_ polarized perpendicular to the molecular plane. The 2s core-hole also creates nearly identical dynamics, but they are difficult to observe due to their very short AM decay time of 0.23 fs. In contrast, the inner-valence hole (3s; green) gives qualitatively different dynamics that do not appreciably oscillate at the femtosecond time scale. We mark the 3s curves in Figure 2 and henceforth as dashed lines to denote that they are inner-valence holes. Also, AM decay is not taken into account for any inner-valence holes due to a lack of literature τ values.

To explain the observed insensitivity to the core hole, Figure 3 shows the hole densities at *t* = 0 for the four different core-holes, plotted at the 0.01 a.u.^–3^ isosurface value. The Cl 1s (a), 2s (b), and 2p (c) hole densities look essentially like those of a Cl atom, and increase in size, as expected. Since the holes are localized and atomic-like, as far as the outer valence electrons are concerned, creating any of these holes reduces the shielding of the nucleus by approximately the same amount. Put another way, all of these core-holes result in a localized ∼+1 charge near the Cl nucleus. This results in dynamics that are insensitive to the core-hole. The 3s hole (d), however, is a molecular orbital hole instead of atomic-like and is delocalized across the entire molecule. The resulting dynamics are thus quasi-static in nature. Additionally, the sudden orbital-hole approximation is generally invalid for molecular orbitals, making these simulations a poor description of the state of a molecule following an ionization pump.

**Figure 3:**
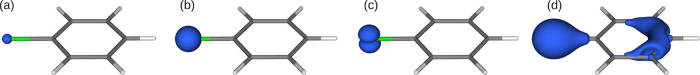
Isosurface plots of the initial hole densities in chlorobenzene for the case of core-hole created in (a) Cl 1s, (b) Cl 2s, and (c) Cl 2p_*y*_. A Cl 3s inner-valence hole (d) is delocalized across the molecule, and due to substantial hybridization with the ring, is not an atomic-like core-hole. This results in qualitatively different dynamics versus and inner-shell hole.

Next, we show dynamics for fluorobenzene and bromobenzene for the case of various core-holes on the halogens. From this point on, we focus on the dynamics in the ring and thus only show the integrated charges on the right half of the benzene ring. This corresponds to integrating over C_3_–C_5_ (see Figure 1 for numbering of carbon). Figure 4a shows the dynamics on the right side of the benzene ring for fluorobenzene after the ionization of a core electron from various core–shells. The F 1s data is damped by the AM decay time of 3.6 fs, and F 2s is undamped. The F 1s triggered dynamics (solid purple) are qualitatively similar to those of the inner-valence (F 2s) triggered case (dashed cyan). This is different from the Cl case, since here the core holes for both 1s and 2s on fluorine are essentially atomic-like rather than molecular orbital like. Since the energy separation between the 2s and 2p orbitals is greater in F than the separation between the 3s and 3p in Cl, the F 2s orbital is less hybridized with the ring, and thus the dynamics triggered by a F 2s core-hole are similar to those of a 1s core-hole, albeit with different magnitudes. Put simpler, the atomic “charge” of F is ∼+1 after the ionization of either a 1s or 2s electron. Figure 4b provides the time-dependent number of electrons on the right half of the ring for bromobenzene. All the inner-shell Br core-holes (1s, 2s, 2p, 3s, 3p) have relatively short AM lifetimes (0.27 0.10, 0.60, 0.15, and 0.20 fs), which lead to no observable coherent charge dynamics. Neglecting AM decay (see SI), all these core-holes give similar dynamics. Also, similar to the F and Cl inner valence case, the 4s core-hole (dashed yellow) does not cause ring dynamics. As discussed with F and Cl, this is due to the valence character of the 4s orbital.

**Figure 4:**
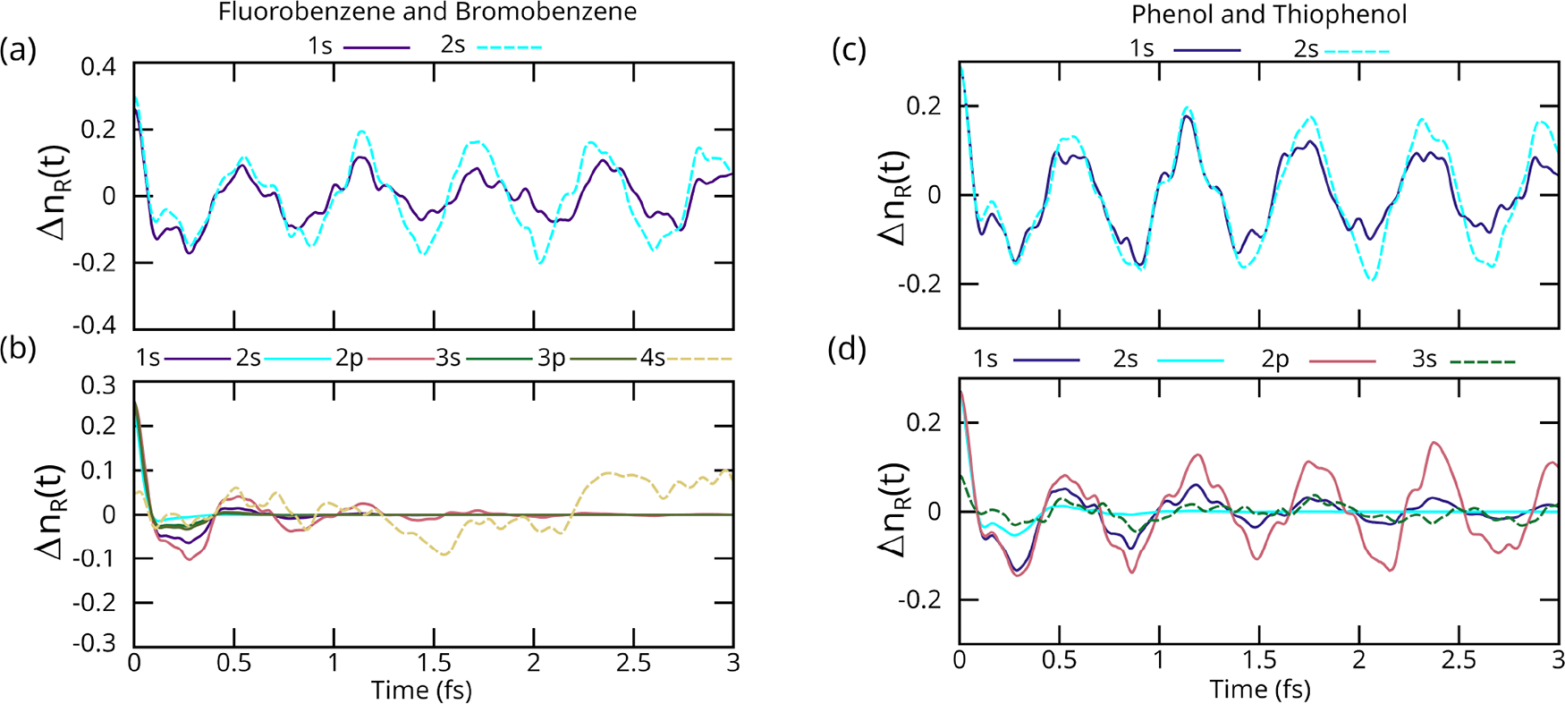
Change in the number of electrons on the right half of the ring for fluorobenzene (a), bromobenzene (b), phenol (c), and thiophenol (d) following different types of core-hole ionization. In phenol analogs, all inner-shell ionization (solid lines) valence dynamics are similar, whereas for inner-valence (dashed), there is no coherent oscillation across the ring. Valence dynamics following inner shell ionization for bromobenzene decay rapidly due to short core-hole life times.

### 3.2 Phenol and Thiophenol

Figure 4c,d shows dynamics for a simple alcohol (phenol) and thiol (thiophenol). Panel (c) shows the charge on the right half of the ring following ionization of an electron from 1s and 2s of the oxygen in phenol. Similar to fluorobenzene, the dynamics triggered by a 2s inner-valence hole (dashed cyan; no AM decay accounted for) is essentially the same as the dynamics triggered by a 1s core hole (solid purple; τ = 4.9 fs). As with F, this is due to the localized shape of a O 2s orbital (i.e., the 2s orbital is sufficiently energetically separated from the valence that it does not hybridize with the ring). Hence, the dynamics on the right half of the ring are similar for O 1s and 2s core-hole triggers. Figure 4d shows the dynamics in thiophenol. The effect of core-hole on the dynamics is similar to the Cl case, where the 1s (solid purple; τ = 1.3 fs) and 2p (solid red; τ = 10 fs) core-holes have atomic-like character and induce similar ring charge oscillations, whereas the 3s (dashed green; no AM accounted for) core-hole does not induce dynamics due to its valence-like character. The lifetime of the core hole for sulfur 2s (solid cyan; τ = 0.27 fs) is relatively small, thus no dynamics are observed.

Comparing the dynamics in the halobenzenes and phenol analogs, all the inner-shell triggered cases give similar femtosecond-scale ring dynamics, which suggests that the main frequency of the dynamics is largely dictated by the ring itself. The character of the excitation was determined via dipole decomposition analysis^71^ to be primarily a π/π* superposition (see SI for details). This is consistent with previous simulations that state that a π/π* transition in nitrosobenzene valence dynamics results from removal of a core electron.^33,34^

### 3.3 Pyridine and Phosphorine

We next present results for pyridine and phosphorine (C_5_H_5_Z, Z = N,P), which are representative of heterocyclic systems. The selected region for charge calculation contains C_3_–C_5_ (see SI). Figure 5a shows the charge on the half of the ring, which is the opposite of a heteroatom. Looking at the pyridine (a) case, the N 1s (solid purple; τ = 7.1 fs) induced dynamics are similar to the N 2s (dashed cyan, no AM accounted for) triggered dynamics, which is consistent with the other two second row element (O, F) cases discussed previously, where the 2s orbital is localized in shape. Figure 5b shows the charge on the opposite half of the ring in the phosphorine. Again, as with the other third row elements (S, Cl), the dynamics induced by 1s (solid purple; τ = 1.4 fs) and 2p (solid red; τ = 18 fs) are similar until the inner-valence 3s orbital (dashed green; no AM accounted for). This is due to the delocalized (molecular-orbital-like) character of these orbitals, for which the sudden approximation is unsuited. Similarly to Cl and S, phosphorus 2s (solid cyan; τ = 0.32 fs) core-holes have a very short lifetime, resulting in unobservable dynamics.

**Figure 5:**
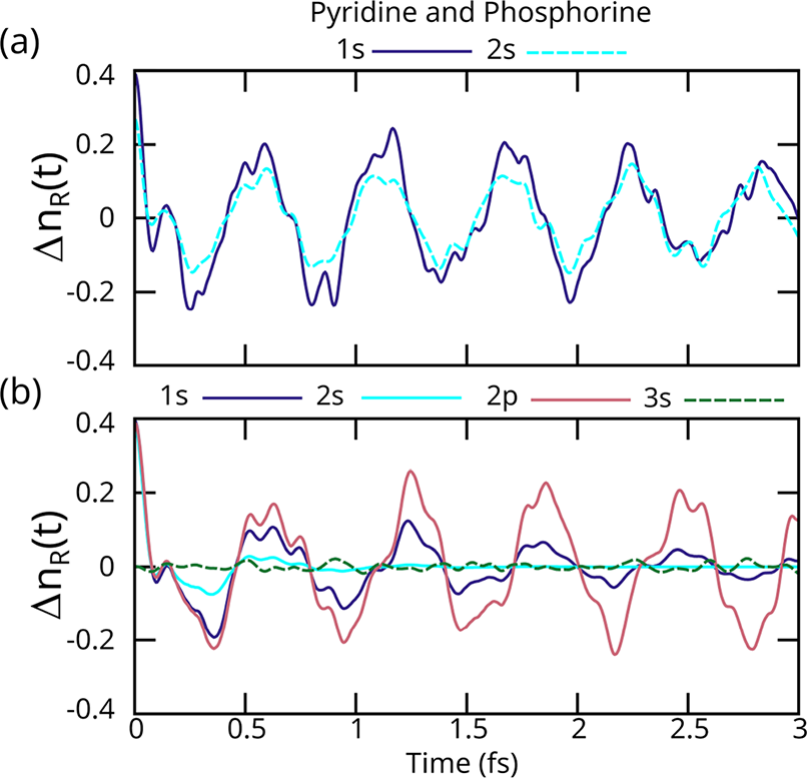
Change in the number of electrons on the right half of the ring for (a) pyridine and (b) phosphorine with different core holes. All the core-holes induce similar dynamics (solid lines), whereas the inner-valence hole (dashed line) induces no coherent valence dynamics.

Comparing across all molecules, the functionalized benzene and heterocyclic results show a slight difference in electron dynamics speeds, with pyridine being faster than phosphorine, phenol slightly faster than thiophenol, and bromobenzene slightly faster than chlorobenzene.

This is a result of different satellite shakeup energies for these molecules, which are related to the π/π* transition, which, as noted above, we determined plays a dominant role using dipole decomposition of the dynamics. We also note that π/π* transitions have long been known to play a dominant role in shakeup in molecules with π systems.^72^ In addition, it is known that the probability of a shake up process is often independent of the core electron ionized, as the valence electrons see similar change in potential around the heteroatom following the ionization.^72^ Likewise, for π-system molecules, the position of the shakeup state is mostly independent of the main ionization channel, as it mainly depends on the π/π* structure of the molecule.^72^ For the single ring molecules studied here, the similarity in the dynamical period is a result of them all belonging to the substituted benzene family. Thus, the observation that dynamics are insensitive to ionization edge (for a particular molecule and particular heteroatom) are consistent with the general characteristics of shakeup satellites.

### 3.4 Chloroazulene

Finally, Figure 6 shows the dynamics in 2-chloroazulene. Azulene is a polycyclic molecule consisting of fused cyclopentadiene and cycloheptatriene moieties and is a building block in many biological chromophores.^73^ In the ground state, this molecule is aromatic-like due to partially positive seven-membered (tropylium) and negative five-membered (cyclopentadienyl) parts. This gives it a substantial dipole moment and allows it to be a strong absorber of visible light. Azulene is thus a good prototypical molecule for understanding attosecond dynamics in conjugated fused-ring systems. Figure 6a,b shows the electron numbers on the seven- and five-membered rings, respectively, following Cl core-hole ionization. For the 2p core-hole case (solid red; τ = 6.4 fs), after the sub 200-as response to the core-hole, there is a ∼0.85 fs period oscillation between the two rings, which involves a max-to-min change in electron number of ∼0.5. Figure 6c shows two snapshots (0.82 and 1.20 fs) of the difference density with respect to the midpoint (*t* = 1 fs) and the isovalue of the positive (blue) 0.001 a.u.^–3^ negative (orange) −0.001 a.u.^–3^. A MO filter is applied to only show π orbitals to give a clear representation of the system, since subtle high frequency oscillations are noticeable when picking particular snapshots of the density to plot. The hole and excess electron densities are localized to the different rings, which means that these dynamics consist of an attosecond electron hopping from one ring to the other. Regarding the effect of core-hole, as with chlorobenzene, 1s (solid purple; τ = 1.2 fs) gives similar π/π* dynamics but with a faster decay. 2s dynamics are not observed due to the 0.23 fs AM decay time. As before, the inner-valence 3s hole (dashed green) does not result in dynamics due to its delocalized shape. Given the relatively slow speed of the dynamics and the large movement of valence electrons involved, chloroazulene and related molecules would make good candidates for soft X-ray pumped experiments. We also used this molecule to assess the effect of DFT exchange-correlation on the dynamics. As shown in the SI in Figure S9, the dynamics are mostly insensitive to DFT functional, with the frequency decreasing somewhat with increasing HF context. Interestingly, Hartree–Fock gives qualitatively similar dynamics to DFT, albeit with a substantially higher frequency. This is presumably related to the well-known tendency of HF to blue-shift excitation energies in TDDFT, due to a lack of electron correlation and general overlocalization of charge.

**Figure 6:**
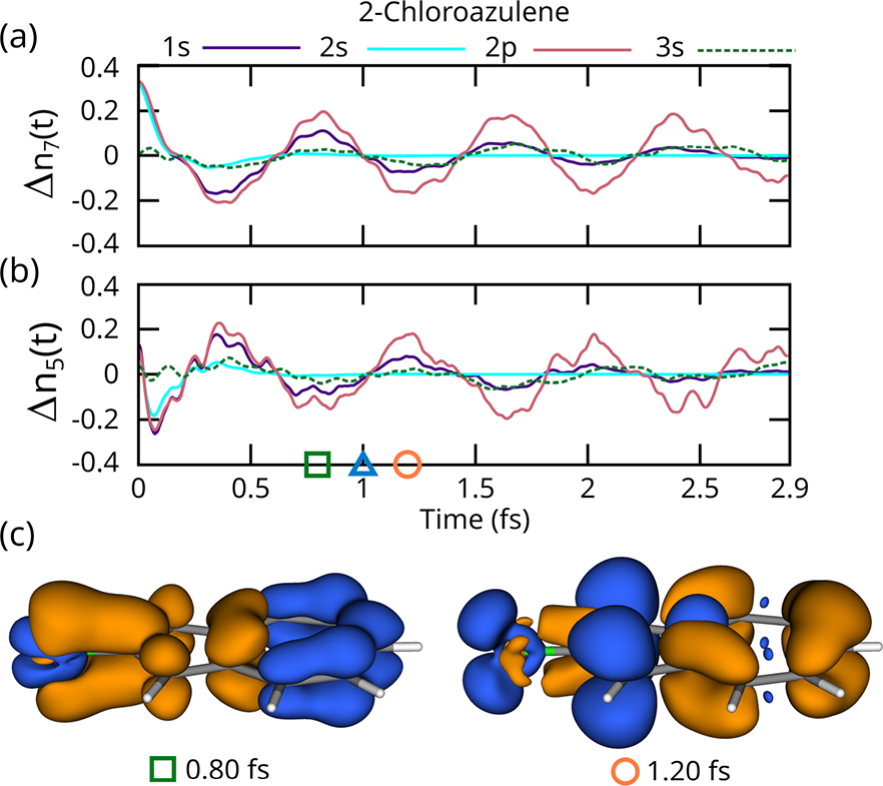
Dynamics in 2-chloroazulene following Cl 2p core-hole ionization. The charges on the seven-membered ring (a) and the five membered ring (b) oscillate with a period of approximately 0.8 fs and with a magnitude of approximately 0.4 electrons. (c) Two snapshots of the difference density (blue isosurface +0.01 a.u., orange isosurface −0.01 a.u.) in the π system with respect to the midpoint of an oscillation (triangle). These show that the extra density moves between the seven- (square, 0.80 fs) and five-membered rings (circle; 1.20 fs).

### 3.5 Validation against XPS Satellites

In order to validate RT-TDDFT for this type of processes, we computed X-ray photoelectron spectra (XPS) shakeup satellite energy shifts for a range of molecules and compared them to experiment. Although not a direct validation of the dynamics (which currently cannot be done due to lack of experiments), shakeup energies are a good test for the ability of TDDFT to correctly predict the correct dynamical time scales. For the purposes of this validation, we took every identifiable experimental satellite peak for each molecule/edge and compared each of those to the corresponding TDDFT peak. In some cases, there are more TDDFT peaks than in the experiment, but it is difficult to comment on this for two reasons. First, each experimental peak is broad enough that there may be multiple satellites under it that cannot be individually distinguished. The locations of the computed TDDFT peaks are generally consistent with this possibility. Second, in the higher energy regions, the TDDFT peaks are too narrow, which is a consequence of our artificial uniform broadening of the peaks. A better description of the continuum may broaden out some of those peaks out entirely. To compute the shift energies, we created a core-hole on the relevant orbital as discussed above, computed the electron number *n*_R_(*t*) on a particular region R, and computed the Fourier transform using a Tukey (cosine-tapered) window, exponential damping, and Padé approximant to the transform.^71^ Each peak in the spectrum corresponds to a distinct dynamical mode, i.e., superposition involving a different satellite. That is, our peak energies, which are on the order of a few eV, can be directly related to experimental shifts in the XPS satellites with respect to the main peak. Since our initial condition is a sudden ionization of a core–electron, these simulations do not respect the correct transition probability/selection rules, and thus, we cannot compare our amplitudes to the experimental ones. Instead, this validation study only tests whether the energies/periods are correct.

This process was performed for a range of molecules and edges for which data was readily available. These include nitrosobenzene (N and O K-edge),^74^ benzonitrile (N K-edge),^75^ pyridine (N K-edge),^76^ 4H-pyran-4-thione (S L_2,3_-edge).^77^ For each of these, density integration region R was chosen to be half of the ring opposite the heteroatom on which the core-hole was created. We also simulated the satellites in *cis*-1,2-dichloroethene (Cl L_1_-edge),^78^ for which *R* was chosen to be a sphere on the opposite Cl, and nitrosyl chloride (Cl L_1_-edge),^79^ for which *R* was a sphere on the O atom. For each of these, the simulation time was 1000 a.u. (24.2 fs) and the exponential damping was τ = 80 a.u. = 1.94 fs. For reference, a subset of the experimental and computed spectra, along with the data table of satellite energies, are shown in SI. Figure 7 shows the computed versus experimental satellite energies for these molecules and edges, where color denotes molecule. Overall, there is good agreement with the experiment, with many satellites being within 0.1 eV of the experiment, and the worst (nitrosobenzene second N K-edge satellite) being 0.6 eV. The mean absolute error (MAE) over 13 values is 0.2 eV. These results show that RT-TDDFT with a sudden core-hole approximation can fairly reliably capture satellite energies. Since these energies are what dictate the dynamics, we can tentatively conclude that RT-TDDFT can capture dynamics involving satellite superpositions. There are some caveats to these results. First, since the sudden ionization is broadband in nature, many high energy superpositions are created, which manifest as small magnitude high frequencies in the dynamics. Second, it will be likely necessary to have some description of the continuum and an explicit laser pulse to get correct XPS intensities.

**Figure 7:**
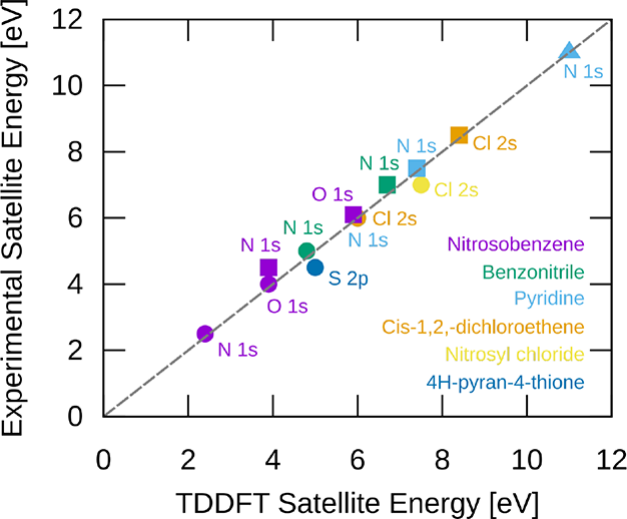
Comparison between the sudden core-hole ionization real-time TD-B3LYP computed XPS satellite shift energies and the experimental ones. Color denotes molecule, and the heteroatoms/edges are labeled with text. Circles denote the first satellite peak in an edge, squares denote the second, and triangles denote the third. Overall, there is good agreement between the computed and experimental satellite shifts.

## 4 Conclusions

In conclusion, we have studied how different core-holes trigger attosecond valence electron dynamics in a set of prototypical functionalized benzene, heterocyclic, and polycyclic molecules. For all molecules studied, we observe, somewhat surprisingly, that the type of core-hole used to launch the dynamics does not significantly affect the dynamics, provided that the core-hole is inner-shell (localized) and not inner-valence (delocalized) and has a sufficiently long Auger-Meiter decay time. For the second row elements (e.g., N, O, F), 2s (inner-valence) core-holes induce π/π* dynamics qualitatively similar to a 1s (inner-shell) core-hole. For the third (P, S, Cl) row elements, inner-valence core-holes (3s) do not induce π/π* oscillations at all. We also observe that the dynamics are largely dictated by the molecule’s valence electronic structure, rather than the core-hole used to trigger them. Specifically, differences in frequency for the various heteroatoms (e.g., phosphorine slower than pyridine) are due to changes in the π/π* transition energy rather than differences in the shapes of the core-holes. This study focuses primarily on the case of functionalized benzene, and the common 0.6 fs period observed for this family of molecules is a consequence of that. These results do not suggest that all molecules have this frequency of core-hole triggered valence dynamics. Chloroazulene, for example, supports much slower dynamics (0.8 fs period) than chlorobenzene. Additionally, these results are limited to the case of a molecule with a single heteroatom, either integrated into, or attached to, the same location in ring. For molecules with different heteroatoms, the dynamics may potentially depend on which heteroatom the core-hole is created on.

These results have many implications for the design of X-ray pump/probe experiments, such as at XFELs, where particular edges or pairs of edges are more practical to work at. Since the dynamics are insensitive to inner-shell core-holes, one has a broad flexibility in which edge to use. Generally speaking, all things equal, using K-edge core-holes for second row elements and L-edge for third row is optimal since these core-holes are localized in space and have long AM lifetimes. The universality of the dynamics opens the door to a wide range of XFEL experiments at different edges, including potentially heavier elements with many inner-shell edges from which to ionize from. Moreover, since dynamics result mostly from the electronic structure of a molecule rather than from the shape of a particular core hole, one can use different heteroatoms/X-ray edges as “tags” for inducing dynamics. These tags can be functionalized at different locations to launch the dynamics from different regions of a molecule. These results are likely to be useful in the design of future XFEL soft X-ray attosecond experiments. Moreover, our results show that sudden core-hole TDDFT gives good agreement with experimental XPS satellite energies, and the data and findings presented here are expected to be useful for more rigorous validation of TDDFT as a means of computing core-hole induced valence dynamics.

## Supplementary Material



## Notes

Molecular geometries, basis set convergence, grid resolution convergence, dipole decomposition of dynamics, table of Auger–Meitner decay times, plots of charge dynamics without AM decay, effect of 2p polarization on dynamics, dependence of dynamics on DFT XC functional, and data table of sudden core-hole TDDFT versus experimental XPS satellite energies (PDF)
