# Supplementary material for: The Effect of Core-Hole Shape on Attosecond Valence Electron Dynamics
Source: J Phys Chem A. 2025 Aug 13;129(34):7742–50. doi: 10.1021/acs.jpca.5c01706 (PMC12400425; doi:10.1021/acs.jpca.5c01706)
Supplement: Supplementary file 1 [file jp5c01706_si_001.pdf]

Supporting Information for: The Effect of Core-Hole Shape on Attosecond Valence Electron Dynamics

Tai Hua,[†] Lucas Kurkowski,[†] and Kenneth Lopata[*,†,‡]

†*Department of Chemistry, Louisiana State University, Baton Rouge, LA,70803*

‡*Center for Computation and Technology, Louisiana State University, Baton Rouge, LA,70803*

E-mail: klopata@lsu.edu

Contents

1 Molecular geometries **3**

2 Basis set convergence **8**

3 Grid resolution convergence **8**

4 Attribution of dynamics via dipole decomposition **9**

5 Auger-Meitner decay times **12**

6 Charge dynamics without Auger-Meitner **14**

7 Chlorobenzene 2p$_x$, 2p$_y$, 2p$_z$ core-hole induced dynamics **17**

8 Dependence of Dynamics on XC Functional **17**

9 XPS and TDDFT Spectra **19**

References **21**

1 Molecular geometries

The def2-TZVP/B3LYP optimized xyz geometries are listed below.

Fluorobenzne (C_6H_5F), positions in Å, long axis is x.

F	-3.43775331	0.00160423	1.19753281
C	-2.08536411	0.00152146	1.19748277
C	-1.41949870	0.00004570	-0.01557121
C	-0.02867287	-0.00034518	-0.00636461
C	0.66835597	0.00020676	1.19737629
C	-0.02857887	0.00175453	2.40117101
C	-1.41940425	0.00281541	2.41048572
H	-1.98435807	-0.00084276	-0.93809451
H	0.50953263	-0.00117091	-0.94570124
H	1.75053170	-0.00059433	1.19733386
H	0.50970009	0.00196853	3.34046618
H	-1.98419020	0.00463759	3.33305291

Chlorobenzene (C_6H_5Cl), positions in Å, long axis is x.

Cl	-3.81464598	0.00070152	1.19755357
C	-2.06743369	0.00159578	1.19748133
C	-1.38587469	0.00003839	-0.01277145
C	0.00438914	-0.00017461	-0.00507700
C	0.70239558	0.00013666	1.19737411
C	0.00448215	0.00156402	2.39988067
C	-1.38577835	0.00328694	2.40768191
H	-1.93665607	-0.00110628	-0.94320569
H	0.54056634	-0.00059629	-0.94564421
H	1.78461088	-0.00067207	1.19733136
H	0.54073112	0.00101263	3.34040754
H	-1.93648644	0.00581433	3.33815784

Bromobenzene (C_6H_5Br), positions in Å, long axis is x.

Br	-3.96543122	0.00121739	1.19755614
C	-2.05270877	0.00160967	1.19748187
C	-1.37338138	0.00008439	-0.01325329
C	0.01759453	-0.00025770	-0.00518530
C	0.71524710	0.00017711	1.19737407
C	0.01768906	0.00171742	2.39998739
C	-1.37328604	0.00294279	2.40816392
H	-1.92042517	-0.00093925	-0.94574037
H	0.55374283	-0.00097619	-0.94601951
H	1.79760365	-0.00067801	1.19733220
H	0.55391141	0.00179764	3.34077980
H	-1.92025599	0.00490576	3.34069306

Phenol (C_6H_5OH), positions in Å, long axis is x.

O	-2.06542497	-0.00168358	1.27976737
C	-0.00119274	0.00000899	2.42171828
C	1.38650946	0.00114000	2.40977401
C	2.08797827	0.00171103	1.20728827
C	1.38398980	0.00113987	0.00927197
C	-0.00664572	0.00000620	0.00780477
C	-0.69903461	-0.00056349	1.21626835
H	-2.43339611	-0.00205972	0.39026566
H	-0.55701733	-0.00044432	3.35026503
H	1.92426422	0.00157555	3.34974620
H	3.16999177	0.00259148	1.20523248
H	1.91548642	0.00157736	-0.93431376
H	-0.55268744	-0.00043182	-0.92979051

Thiophenol (C_6H_5SH), positions in Å, long axis is x.

C	-0.69802192	-0.00416517	1.19232867
C	0.01332476	-0.00398013	-0.00814087
C	1.40260260	-0.00458153	0.00580067
C	2.09782865	0.00059937	1.20969955
C	1.38829043	0.00164501	2.40573041
C	-0.00029988	-0.00322951	2.40196173
S	-2.47104533	0.00863458	1.26270525
H	-0.51401293	-0.00102783	-0.95379053
H	1.94135920	-0.00371717	-0.93377213
H	3.18004940	0.00370439	1.21587814
H	1.91687435	0.00373118	3.35099828
H	-0.54285392	-0.00793519	3.33945797
H	-2.69060540	-0.09943801	-0.05629714

Pyridine (C_5H_5N), positions in Å, long axis is x.

N	-0.67747996	-0.00000418	1.18769818
C	0.01464377	0.00000262	0.04794897
C	1.40424202	-0.00000002	-0.00597208
C	2.11347734	0.00000406	1.18769809
C	1.40424233	0.00000115	2.38136848
C	0.01464392	-0.00000078	2.32744763
H	-0.56938543	0.00000070	-0.86678821
H	1.91296013	0.00000162	-0.96135231
H	3.19651168	0.00000094	1.18769821
H	1.91296042	0.00000339	3.33674881
H	-0.56938620	-0.00000005	3.24218429

Phosphorine (C_5H_5P), positions in Å, long axis is x.

P	1.09851450	-0.04062648	1.40666726
C	0.02692140	-0.00077192	0.04383239
C	-1.35772299	0.04994712	0.11422263
C	-2.05943476	0.07604947	1.31600014
C	-1.42764512	0.05293641	2.55591694
C	-0.04944431	0.00162382	2.70571379
H	0.48778851	-0.01739354	-0.93834818
H	-1.92637789	0.07056819	-0.80999885
H	-3.14140205	0.11596992	1.28522216
H	-2.04826766	0.07638384	3.44596826
H	0.35406390	-0.01359853	3.71273785

Benzonitrile (C_7H_5N), positions in Å.

H	-1.99129690	-0.04558207	-0.94987517
H	0.48250764	0.01312711	-0.94597822
C	-1.44500872	-0.03185056	-0.01642722
C	-0.05855053	0.00116065	-0.00866957
N	-4.72807734	-0.11210128	1.19261980
C	-3.57596115	-0.08306806	1.19368637
C	-2.14516032	-0.04764853	1.19500491
C	0.63442114	0.01859926	1.19755488
H	1.71661904	0.04401542	1.19855150
C	-0.06081785	0.00349359	2.40250434
C	-1.44728967	-0.02950302	2.40771949
H	0.47848132	0.01727224	3.34080235
H	-1.99533337	-0.04142413	3.34016303

Nitrosobenzene (C_6H_5NO), positions in Å.

H	1.9219834227	-0.2052209193	-0.9369146597
H	-0.5690458705	0.0607482748	-0.9192194034
C	1.3807889348	-0.1474611958	-0.0014080302
C	0.0000000000	0.0000000000	0.0000000000
N	-2.1082129121	0.2251622101	1.1042925305
C	2.0647410352	-0.2206079402	1.2072557356
H	3.1416138188	-0.3357110901	1.2096530773
C	-0.6816012737	0.0728517907	1.2121948208
O	-2.7081494976	0.2900998223	2.1536569394
C	1.3753428899	-0.1470192697	2.4204189084
C	-0.0000000000	-0.0000000000	2.4292955124
H	-0.5625427933	0.0600762105	3.3513918182
H	1.9211463778	-0.2054803005	3.3537334768

Cis-1,2-dichloroethene ($C_2H_2Cl_2$), positions in Å.

Cl	0.46132445	0.00116080	-0.31193502
Cl	-2.85131753	0.00354868	-0.31193335
C	-0.53235196	0.00045821	1.09332909
C	-1.85763869	0.00141995	1.09335722
H	0.01550466	-0.00087869	2.02449189
H	-2.40552092	0.00089104	2.02450182

Nitrosyl chloride (NOCl), positions in Å.

N	-0.78203804	-0.54528805	-0.00000000
O	-1.56351293	0.26864886	-0.00000000
Cl	1.09235096	0.12473919	0.00000000

4H-pyran-4-thione (C_5H_4OS), positions in Å.

H	1.85786969	-0.00017473	-0.99463186
H	-0.63322798	-0.00029487	-0.84564232
C	1.36254698	-0.00000732	-0.03410688
C	0.02002013	-0.00011603	0.01441471
C	2.15814596	0.00015538	1.17199985
S	3.81327265	-0.00011149	1.17199995
O	-0.68273611	-0.00008021	1.17200020
C	0.02002030	0.00018115	2.32958538
C	1.36254718	0.00039014	2.37810666
H	-0.63322805	0.00012648	3.18964233
H	1.85786924	0.00058150	3.33863200

2-Chloroazulene ($C_{10}H_7Cl$), positions in Å, long axis is x.

Cl	-4.94045001	-0.00000077	1.60601538
C	-2.42662373	0.00000722	2.76042876
C	-1.08739684	0.00000254	2.35320376
C	0.01640724	0.00000219	3.19581330
C	1.36859022	-0.00000523	2.86674550
C	1.96030886	0.00000003	1.60601655
C	1.36859040	0.00000509	0.34528750
C	0.01640744	-0.00000182	0.01622057
C	-1.08739663	-0.00000273	0.85883021
C	-2.42662327	-0.00000720	0.45160465
C	-3.21523081	-0.00000025	1.60601650
H	-2.78750757	0.00001464	3.77644932
H	-0.21145728	0.00000091	4.25793478
H	2.05479667	-0.00000601	3.70618685
H	3.04541286	0.00000015	1.60601700
H	2.05479723	0.00000663	-0.49415333
H	-0.21145773	-0.00000126	-1.04590056
H	-2.78750677	-0.00001492	-0.56441598

2 Basis set convergence

In this section, we show the basis sets convergence of the dynamics for chlorobenzene. Fig. S1 shows the dipole moment following Cl K-edge ionization for the following basis sets: def2-SVP, def2-TZVP, def2-QZVP, and a mixed basis consisting of def2-TZVP for C and H, and def2-QZVP for chlorine. The slower ($\sim$0.6 fs period) mode is essentially converged with the mixed basis and QZVP basis. We henceforth used the mixed basis for all calculations. There are slight differences in the high frequency modes, but they are not the focus of this work.

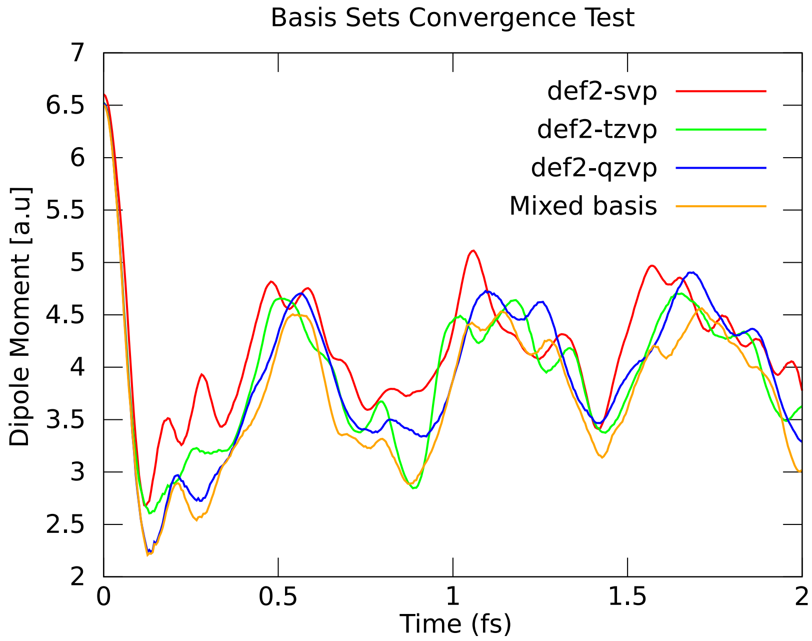

Fig. S1: Convergence of the dipole moment with basis set for chlorobenzene and a Cl 1s core-hole. The main (slow) electron dynamics mode is converged with the mixed (TZVP C/H, QZVP Cl) basis.

3 Grid resolution convergence

Fig. S2 shows the computed change in number of electrons $\Delta n_R(t)$ on the right half of the ring for chlorobenzene following Cl K-edge ionization, for five different grid resolutions. All

simulations had a minimum buffer of 3.0 Å from each atom. The region was taken to be $x \in (-\infty, x_0)$, $y \in (-\infty, \infty)$, $z \in (-\infty, \infty)$, where x_0 is the x-midpoint between Cl and its neighboring C. For the left half of the ring, $n_\mathrm{L}(t)$, the region was $x \in (x_0, x_1)$, $y, z \in (-\infty, \infty)$, where x_1 is the x-midpoint of the ring. Finally, the number of electrons on the right half of the ring, $n_\mathrm{R}(t)$ was integrated over the region $x \in (x_1, \infty)$, $y, z \in (-\infty, \infty)$. Based on these results, to ensure convergence with grid, we used a resolution of $\Delta x = \Delta y = \Delta z = 0.035$ Å for all subsequent simulations. For the chlorobenzene molecule, for example, this corresponds to a $332 \times 173 \times 295$ grid.

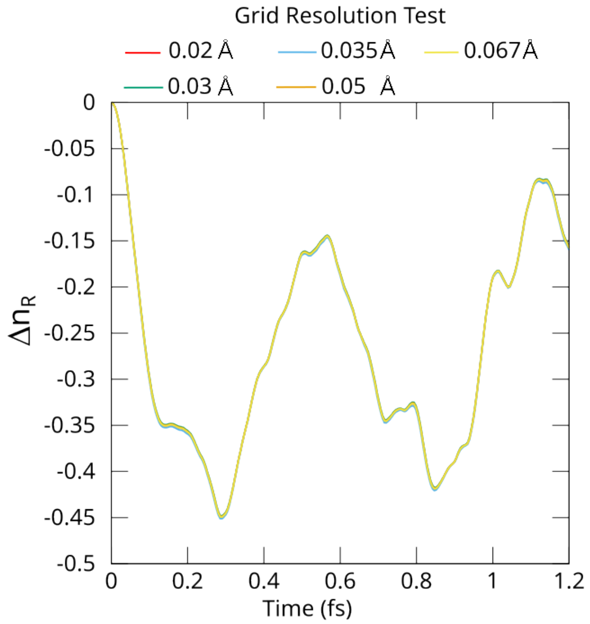

Fig. S2: Change in charge on the right half of chlorobenzene for various grid resolutions. All resolutions are in Å.

4 Attribution of dynamics via dipole decomposition

To determine which molecular orbitals are predominantly involved in the dynamics, we calculated the time-dependent dipole pair contribution.[1,2] Briefly, this involves computing the MO dipole tensor:

$$\mathbf{M}^d_{ij}(t) = \sum_{k=1}^{m} \mathbf{D}_{ik}\mathbf{P}_{kj}(t) \tag{S1}$$

where $d = x, y, z$, $\mathbf{D}$ is the transition dipole matrix in the MO basis, and $\mathbf{P}(t)$ is the density matrix in the MO basis. See Ref. 1 for details about change of bases in electronic structure codes. The time-dependent dipole is then:

$$\mu^d(t) = \text{Tr}\left[\mathbf{M}^d_{ij}(t)\right] \tag{S2}$$

The contributions to the dipole moment from occupied/virtual pairs of MOs is given by:

$$\mu_{ia}(t) = \mathbf{D}^{MO}_{ia}\mathbf{P}^{MO}_{ai}(t) + \mathbf{D}^{MO}_{ai}\mathbf{P}^{MO}_{ia}(t) \tag{S3}$$

Looking at the magnitude of the contributions of i/a pairs in eq. S3 for a particular frequency, it can be used to quantify the orbitals involved in the dynamics.

Fig. S3(a) presents the MO occupation number of chlorobenzene following a 1s core-hole. Upon the ionization of a 1s electron, many occupied and virtual states changes in occupation number, specifically, the occupation number of MO 26 being significantly decreased and MO 31 increased. As shown in Fig. S3(b), however, the dipole contribution of the MO 26/31 pair has little contribution to the total dipole (c).

Fig. S3(d-h) shows a selection of pair dipole contributions from various occupied orbitals to the LUMO or LUMO+1, along with their Fourier transforms. The FFT shows a dominant frequency around $\omega = 0.27$ a.u. ($T = 23.3$ a.u. $= 0.57$ fs), which corresponds to the slow oscillation in the ring dynamics. Looking at the various MO dipole pairs, pair 29 (π) / 31 (π^*) contains this frequency and has a high amplitude, thus we conclude that the dynamics are predominantly a π/π^* superposition. This is consistent with Kuleff et.al,[3] who showed the $\pi \rightarrow \pi^*$ transition is important for the valence dynamics upon the ionization of a core electron.[3]

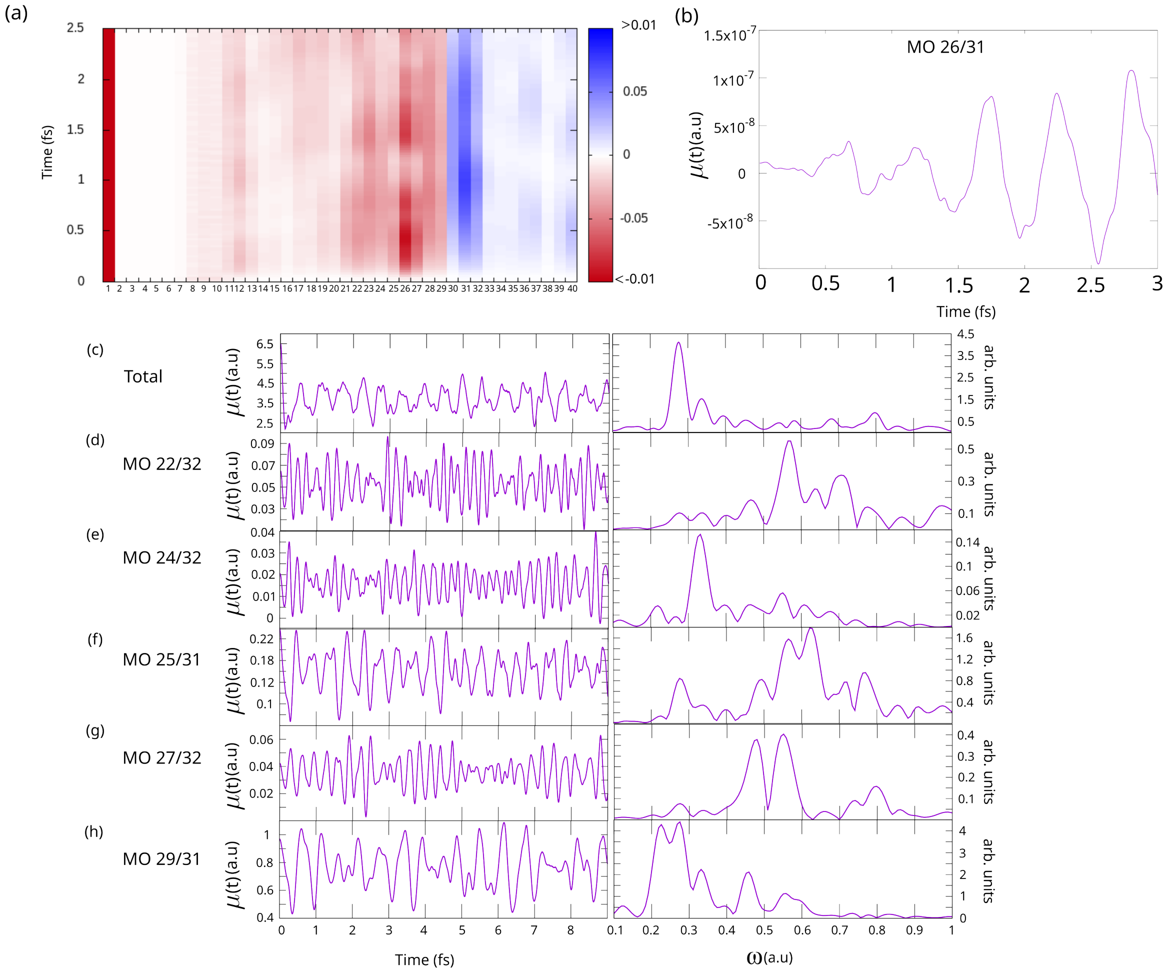

Fig. S3: (a) The chlorobenzene time dependent change in MO occupation number. (b) The occupied MO 26 / virtual MO 31 dipole pair shows little coherence and thus does not contribute to the dynamics. (c) The total dipole moment and its Fourier transform. (d-h) MO pair dipoles and their Fourier transforms. MO pair 29 has a large peak at the main dynamical frequency, which means the dynamics are largely π/π^* in nature.

5 Auger-Meitner decay times

Tab. S1 shows the literature calculated decay times for various core holes.[4] The creation of a core vacancy in an atom causes two types of transitions: radiative and nonradiative. In the radiative transition, an outer shell electron filled the vacancy and emits x-ray radiation. This is generally a slow process. In the nonradiative transition, an outer electron fills the vacancy, and the extra energy results in the ejection of a valence electron. If the vacancy is filled by an electron from the same shell, then the process is a Coster-Kronig transition, whereas Auger-Meitner refers to a vacancy filled by an electron from a different shell. The mean lifetime τ can be calculated as $\tau = \frac{\tau_{\text{rad}}\tau_{\text{nonrad}}}{\tau_{\text{rad}}+\tau_{\text{nonrad}}}$. In practice, since the nonradiative processes are generally orders of magnitude faster than the radiative, $\tau \simeq \tau_{\text{rad}}$. As discussed in the manuscript, in order to phenomenologically account for decay, we exponentially damp our computed charges by the mean lifetime τ.

Table S1: Simulated binding energies (E_b) and decay lifetimes (τ) for the core-holes used in this study. All data taken from Ref. 4. Mean lifetimes include both radiative and non-radiative (Auger-Meitner and/or Coster-Kronig) decay pathways. Since we do not incorporate spin-orbit coupling, the L_2 edge lifetime is chosen for our 2p calculations.

Element	Core-hole	E_b [eV]	τ_{rad} [fs]	τ_{nonrad} [fs]	τ [fs]
N	K	404.9	2.0×10^3	7.1	7.1
	L_1	23.1	–	–	–
O	K	537.28	8.0×10^2	5.0	4.9
	L_1	29.23	–	–	–
F	K	688.37	3.8×10^2	3.6	3.6
	L_1	35.93	–	–	–
P	K	2130.4	2.3×10^1	1.5	1.4
	L_1	187.15	1.6×10^4	0.32	0.32
	L_2	139.14	8.3×10^4	18	18
	L_3	138.18	8.2×10^4	18	18
	M_1	17.21	–	–	–
S	K	2455.90	1.7×10^1	1.4	1.3
	L_1	255.97	8.5×10^3	0.27	0.27
	L_2	172.73	5.4×10^4	10	10
	L_3	171.4	5.4×10^4	10	10
	M_1	20.95	–	–	–
Cl	K	2804.90	1.2×10^1	1.3	1.2
	L_1	268.05	4.7×10^3	0.23	0.23
	L_2	209.48	3.8×10^4	6.4	6.4
	L_3	207.7	3.7×10^4	6.3	6.3
	M_1	24.84	–	–	–
Br	K	13435	4.4×10^{-1}	0.72	0.27
	L_1	1769.20	4.7×10^1	0.10	0.10
	L_2	1602.10	3.2×10^1	0.60	0.60
	L_3	1553.60	3.3×10^1	0.62	0.60
	M_1	254.49	6.2×10^3	0.15	0.15
	M_2	191.82	3.0×10^3	0.18	0.20
	M_3	184.78	3.4×10^3	0.20	0.20
	N_1	24.2	–	–	–

6 Charge dynamics without Auger-Meitner

In this section, we present the valence dynamics after the ionization of a core electron without accounting for Auger decay.

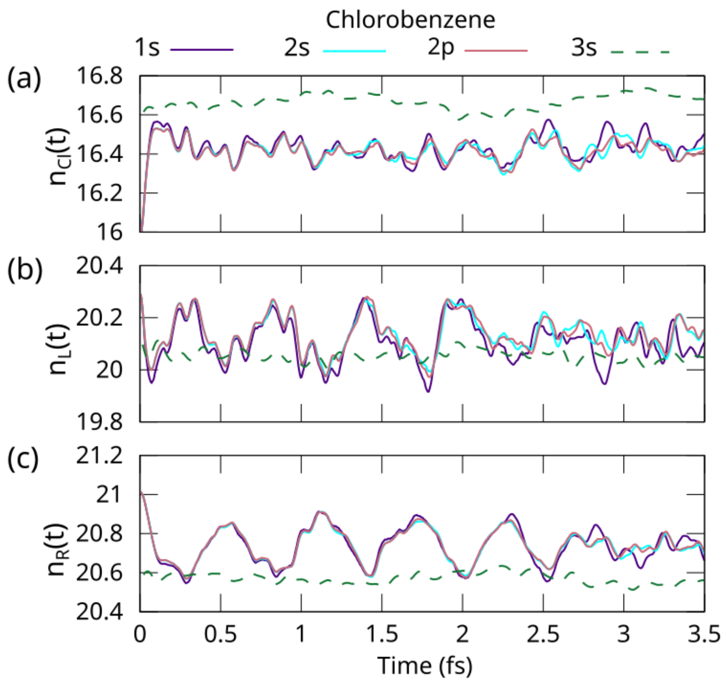

Fig. S4: Time-dependent change in number of electrons on three regions of chlorobenzene following different core-hole ionization triggers: (a) around the Cl, (b) on the left half of the benzene ring, and (c) on the right half of the ring. On each plot, solid lines denote inner-shell ionization, and dashed-lines denote inner-valence. No AM decay was accounted for.

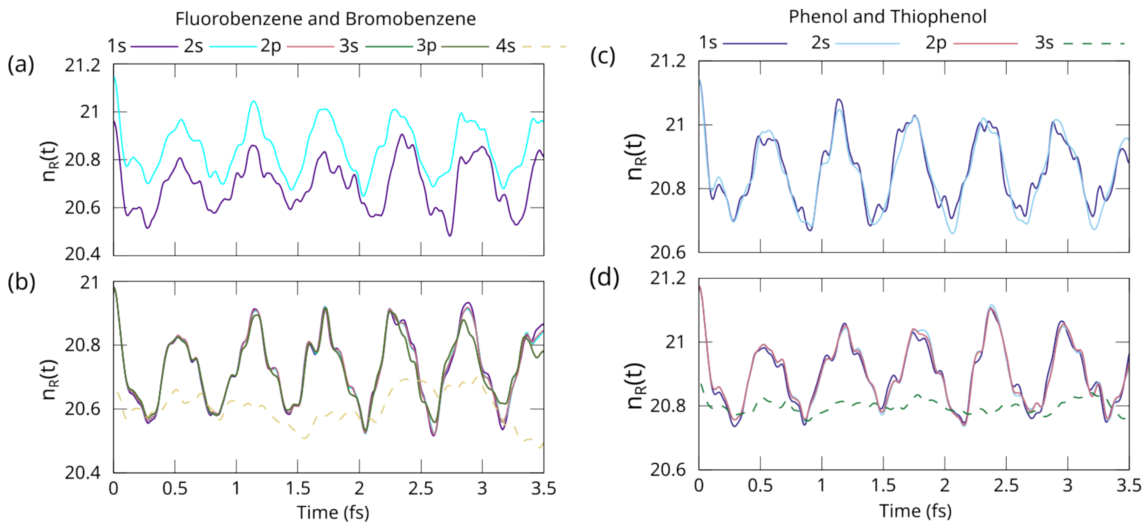

Fig. S5: Change in number of electrons on the right half of the ring for fluorobenzene (a), bromobenzene (b), phenol (c) and thiophenol (d) following different types of core-hole ionization. In phenol analogs, all inner-shell ionization (solid lines) valence dynamics are similar, whereas for inner-valence (dashed) there is no coherent oscillation across the ring. No valence dynamics following inner shell ionization for bromobenzene due to short core-hole life time. No AM decay was accounted for.

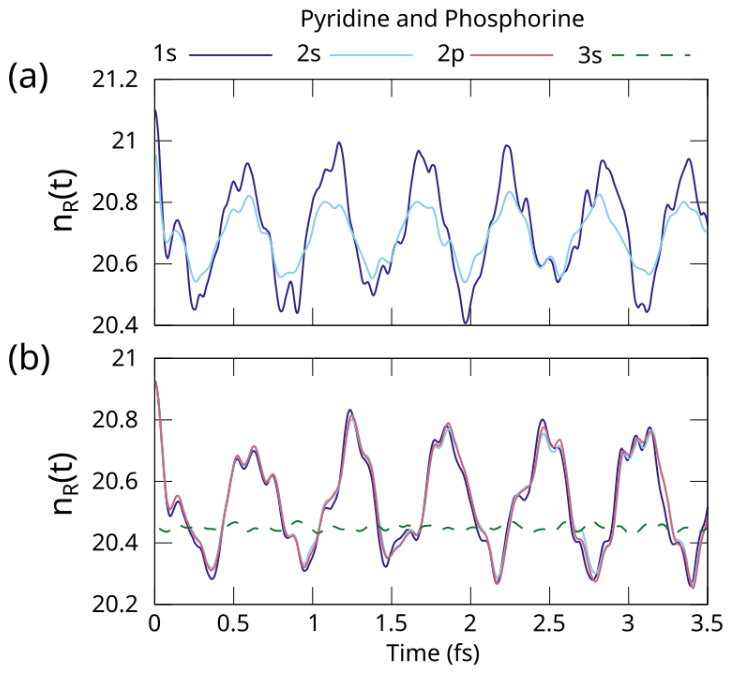

Fig. S6: Change in number of electrons on the right half of the ring for (a) pyridine and (b) phosphorine with different core holes. All the core-holes induce similar dynamics (solid lines), whereas the inner-valence hole (dashed line) induces no coherent valence dynamics. No AM decay was accounted for.

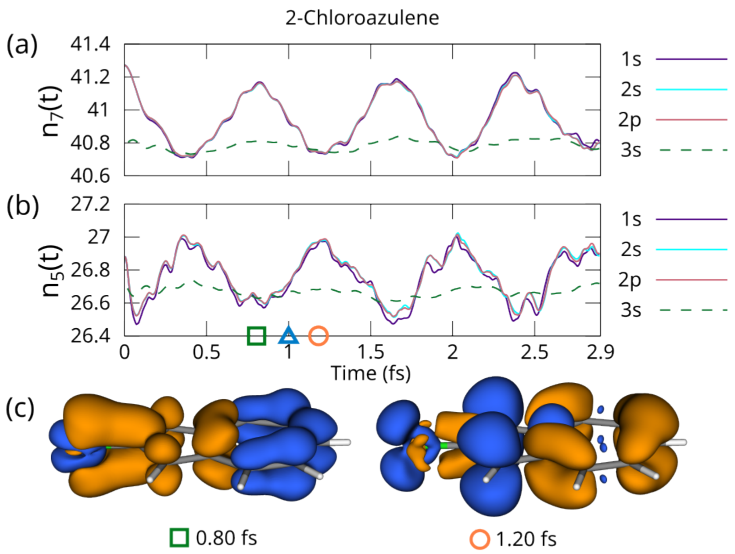

Fig. S7: Dynamics in 2-chloroazulene following Cl 2p core-hole ionization. The charges on the seven-membered ring (a) and the five-membered ring (b) oscillate with a period of approximately 0.8 fs and with a magnitude of approximately 0.4 electrons. Panel (c) shows two snapshots of the difference density in the π system with respect to the midpoint of an oscillation (triangle). These show that the extra density moves between the seven (square, 0.80 fs) and five-membered rings (circle; 1.20 fs). No AM decay was accounted for.

7 Chlorobenzene $2p_x$, $2p_y$, $2p_z$ core-hole induced dynamics

We next present the dynamics in chlorobenzene for various 2p core-holes. Fig. S8 shows the $\Delta n(t)$ for chlorine (a), left benzene (b) and right benzene (c) with different 2p polarization. For these plots, no AM decay was accounted for. The polarization of the core hole has little effect on the ring dynamics. Based on this, in the manuscript, for all 2p core-holes we used a $2p_y$ ($\perp$ to plane) hole.

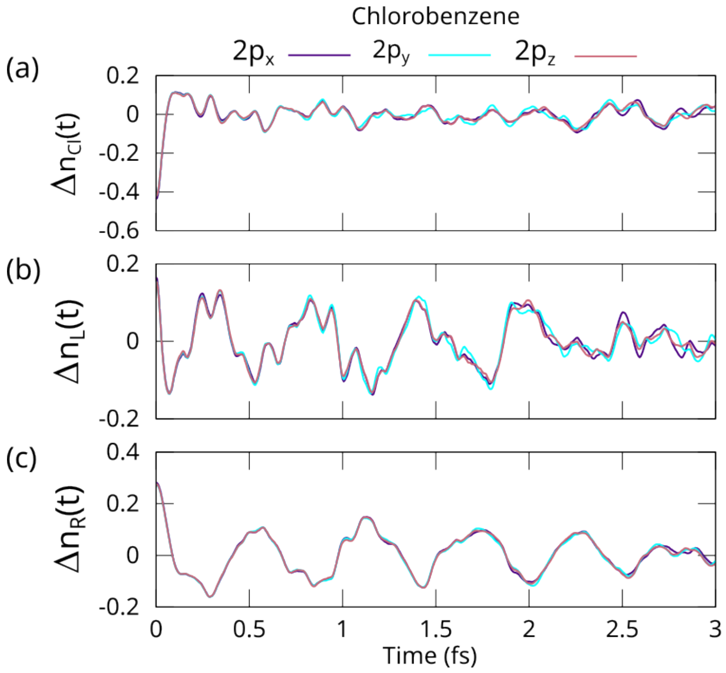

Fig. S8: Change in number of electrons on the chlorine (a), left (b) and right half of benzene (c). All the polarized core-holes induce similar dynamics.

8 Dependence of Dynamics on XC Functional

In this section, we present the Cl 2p initiated dynamics in chloroazulene using a range of DFT functionals. Fig. S9 shows the $\Delta n_7(t)$ computed along with TD HF, LDA, PBE, PBE0, B3LYP and CAM-B3LYP.

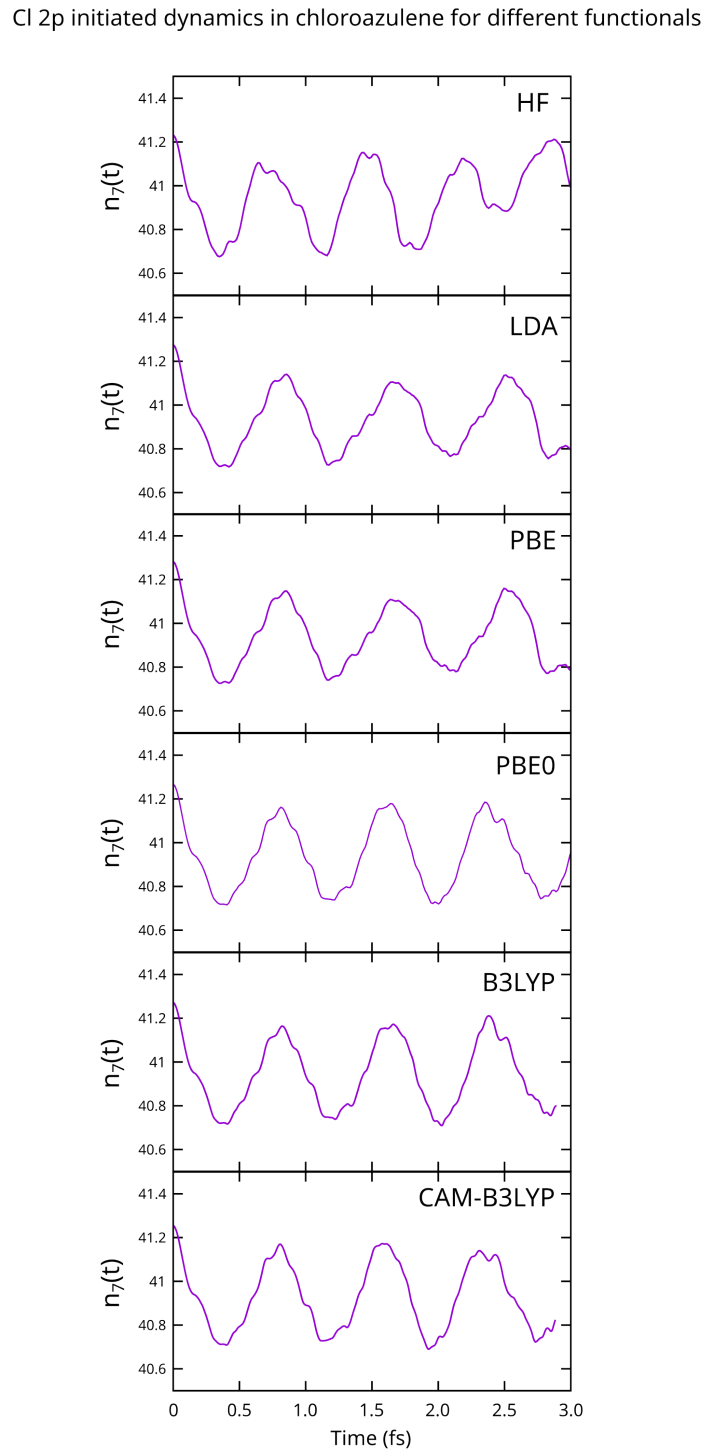

Fig. S9: Time-dependent electron norm on the seven-membered ring in chloroazulene following Cl 2p ionization, computed using TD Hartree-Fock (HF) and TDDFT using various DFT functionals. Qualitatively, the TDDFT dynamics are insensitive to DFT functional, with the frequency decreasing somewhat with increasing HF context. Range-separated (CAM-B3LYP) and global hybrid (B3LYP) have similar dynamics.

9 XPS and TDDFT Spectra

Fig. S10 shows a few experimental XPS spectra, along with the TDDFT computed charge dynamics transforms. As discussed in the main manuscript, the sudden core-hole approximation does not give the correct peak amplitudes, but the frequencies agree well with experiment. The low energy peak in nitrosyl chloride (b) is likely due to a low frequency mode in the dynamics that would require a longer simulation time to resolve. The corresponding peak energies are given in Tab. S2.

Table S2: TDDFT computed shake-up satellite energy shifts compared to experimentally measured ones, for a selection of molecules and edges. The TDDFT (TD-B3LYP) values were computed from the Fourier transform of the time-dependent charge on a region of the molecule following sudden ionization from the specified element/edge.

**Molecule**	**Edge**	**TDDFT (eV)**	**Experiment (eV)**	**Expt. Ref.**
Nitrosobenzene	N 1s	2.4	2.5	9
		3.9	4.5	
	O 1s	3.9	4.0	
		5.9	6.1	
Benzonitrile	N 1s	4.8	5.0	10
		6.7	7.0	
Pyridine	N 1s	6.0	6.0	8
		7.4	7.5	
		11.0	11.0	
Cis-1,2,-dichloroethene	Cl 2s	6.0	6.0	11
		8.4	8.5	
Nitrosyl chloride	Cl 2s	7.5	7.0	6
4H-pyran-4-thione	S 2p	5.0	4.5	7

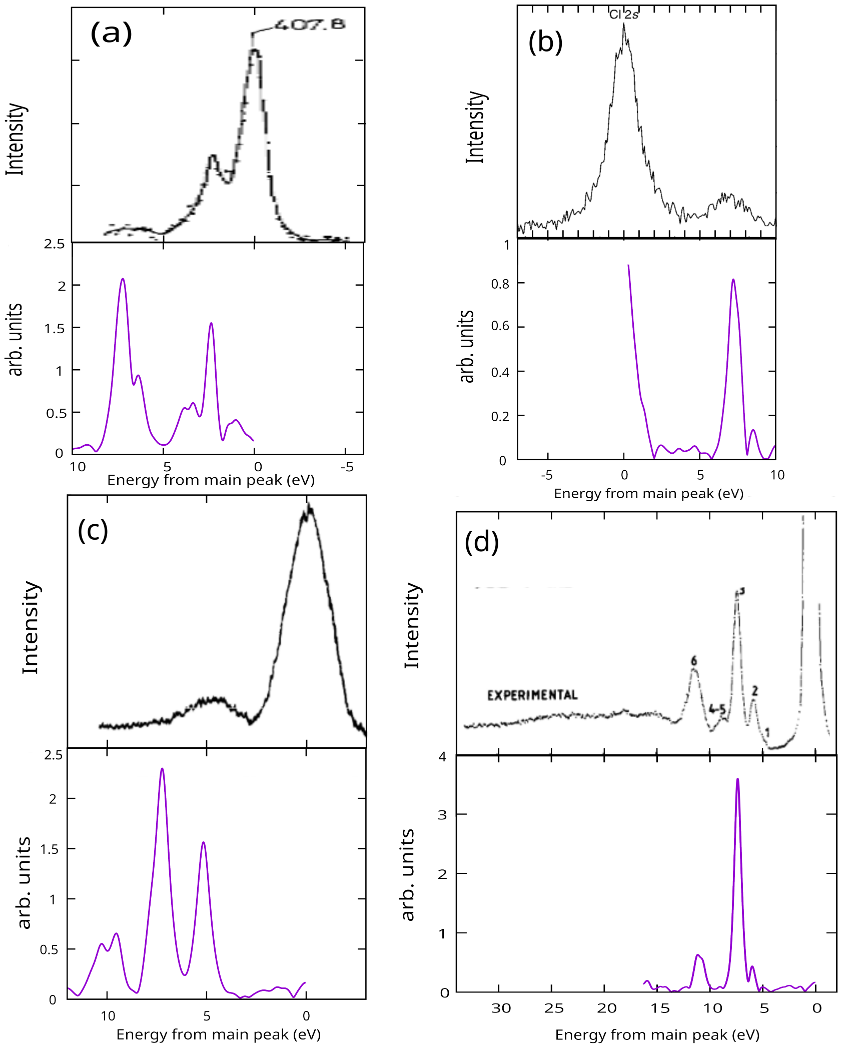

Fig. S10: Experimental XPS spectra (black) along with transforms of the TDDFT computed charge number (purple) for (a) nitrosobenzene N 1s. Experimental curve is reproduced with permission from Ref. 5. Copyright 1987 Elsevier; (b) nitrosyl chloride Cl 2s. Experimental curve is reproduced with permission from Ref. 6. Copyright 2015 Royal Society of Chemistry.; (c) 4H-pyran-4-thione S 2p. Experimental curve is reproduced with permission from Ref. 7. Copyright 1980 Elsevier.; (d) pyridine N 1s. Experimental curve is reproduced with permission from Ref. 8. Copyright 1991 Elsevier.

References

(1) Bruner, A.; LaMaster, D.; Lopata, K. Accelerated broadband spectra using transition dipole decomposition and Padé approximants. *J. Chem. Theory Comput.* **2016**, *12*, 3741–3750.

(2) Yang, M.; Sissay, A.; Chen, M.; Lopata, K. Intruder peak-free transient inner-shell spectra using real-time simulations. *J. Chem. Theory Comput.* **2022**, *18*, 992–1002.

(3) Kuleff, A. I.; Kryzhevoi, N. V.; Pernpointner, M.; Cederbaum, L. S. Core ionization initiates subfemtosecond charge migration in the valence shell of molecules. *Phys. Rev. Lett.* **2016**, *117*, 093002.

(4) Perkins, S. T.; Cullen, D. E.; Chen, M. H.; Rathkopf, J.; Scofield, J.; Hubbell, J. H. *Tables and graphs of atomic subshell and relaxation data derived from the LLNL Evaluated Atomic Data Library (EADL), Z = 1–100*; 1991.

(5) Sjögren, B.; Freund, H.-J.; Salaneck, W.; Bigelow, R. Core ionization of nitrosobenzene-dimer compounds: phenazon-di-N-oxide. *chem. phys.* **1987**, *118*, 101–112.

(6) Schio, L.; Li, C.; Monti, S.; Salén, P.; Yatsyna, V.; Feifel, R.; Alagia, M.; Richter, R.; Falcinelli, S.; Stranges, S.; others NEXAFS and XPS studies of nitrosyl chloride. *Physical Chemistry Chemical Physics* **2015**, *17*, 9040–9048.

(7) Colonna, F. P.; Distefano, G.; Guerra, M.; Jones, D. Photoelectron (He (I), He (II) and X-ray) spectroscopy of γ-pyrone and its related sulphur derivatives: valence and core ionization energies and shake-up satellites. *Journal of Electron Spectroscopy and Related Phenomena* **1980**, *18*, 309–328.

(8) Keane, M.; de Brito, A. N.; Correia, N.; Svensson, S.; Lunell, S. Experimental and theoretical study of the N1s and C1s shake-up satellites in pyridine and aniline. *Chemical physics* **1991**, *155*, 379–387.

(9) Distefano, G.; Guerra, M.; Jones, D.; Modelli, A.; Colonna, F. P. Experimental and theoretical study of intense shake-up structures in the XPS spectra of nitrobenzenes and nitrosobenzenes. *Chemical Physics* **1980**, *52*, 389–398.

(10) Ohta, T.; Fujikawa, T.; Kuroda, H. Core-electron spectra of mono-substituted benzenes obtained by the gas-phase X-ray photoelectron spectroscopy. *Bulletin of the Chemical Society of Japan* **1975**, *48*, 2017–2024.

(11) Berndtsson, A.; Basilier, E.; Gelius, U.; Hedman, J.; Klasson, M.; Nilsson, R.; Nordling, C.; Svensson, S. Ethene and the Chloroethenes Studied by ESCA. *Physica Scripta* **1975**, *12*, 235.
